# Optimal acquisition scheme for flow‐compensated intravoxel incoherent motion diffusion‐weighted imaging in the abdomen: An accurate and precise clinically feasible protocol

**DOI:** 10.1002/mrm.27990

**Published:** 2019-09-30

**Authors:** Oliver J. Gurney‐Champion, Susanne S. Rauh, Kevin Harrington, Uwe Oelfke, Frederik B. Laun, Andreas Wetscherek

**Affiliations:** ^1^ Joint Department of Physics The Institute of Cancer Research and The Royal Marsden NHS Foundation Trust London United Kingdom; ^2^ Institute of Radiology University Hospital Erlangen, Friedrich‐Alexander‐Universität Erlangen‐Nürnberg (FAU) Erlangen Germany; ^3^ Targeted Therapy team The Institute of Cancer Research and The Royal Marsden NHS Foundation Trust London United Kingdom

**Keywords:** Diffusion‐weighted MRI, intravoxel incoherent motion, perfusion, quantitative MRI

## Abstract

**Purpose:**

Flow‐compensated (FC) diffusion‐weighted MRI (DWI) for intravoxel‐incoherent motion (IVIM) modeling allows for a more detailed description of tissue microvasculature than conventional IVIM. The long acquisition time of current FC‐IVIM protocols, however, has prohibited clinical application. Therefore, we developed an optimized abdominal FC‐IVIM acquisition with a clinically feasible scan time.

**Methods:**

Precision and accuracy of the FC‐IVIM parameters were assessed by fitting the FC‐IVIM model to signal decay curves, simulated for different acquisition schemes. Diffusion‐weighted acquisitions were added subsequently to the protocol, where we chose the combination of b‐value, diffusion time and gradient profile (FC or bipolar) that resulted in the largest improvement to its accuracy and precision. The resulting two optimized FC‐IVIM protocols with 25 and 50 acquisitions (FC‐IVIM_opt25_ and FC‐IVIM_opt50_), together with a complementary acquisition consisting of 50 diffusion‐weighting (FC‐IVIM_comp_), were acquired in repeated abdominal free‐breathing FC‐IVIM imaging of seven healthy volunteers. Intersession and intrasession within‐subject coefficient of variation of the FC‐IVIM parameters were compared for the liver, spleen, and kidneys.

**Results:**

Simulations showed that the performance of FC‐IVIM improved in tissue with larger perfusion fraction and signal‐to‐noise ratio. The scan time of the FC‐IVIM_opt25_ and FC‐IVIM_opt50_ protocols were 8 and 16 min. The best in vivo performance was seen in FC‐IVIM_opt50_. The intersession within‐subject coefficients of variation of FC‐IVIM_opt50_ were 11.6%, 16.3%, 65.5%, and 36.0% for FC‐IVIM model parameters diffusivity, perfusion fraction, characteristic time and blood flow velocity, respectively.

**Conclusions:**

We have optimized the FC‐IVIM protocol, allowing for clinically feasible scan times (8‐16 min).

## 1 INTRODUCTION

Capillary perfusion plays a large role in many major diseases, including cancer, and is prognostic for many indications.^1^, ^2^ MRI offers several approaches to assessing perfusion, of which the intravoxel incoherent motion (IVIM) model fit to diffusion‐weighted MRI (DWI) data is a promising example without contrast injection. The IVIM model is a two‐compartment model for DWI,^3^ in which signal decay is described by a tissue compartment and a perfusion compartment. The latter describes incoherent motion, which is often attributed to blood flow, but can also be related to other sources.^4^

Almost all IVIM studies assume the pseudo‐diffusion limit of the IVIM model, which requires the diffusion time (*T*) to be several times larger than the characteristic time scale (τ) of the capillary perfusion.^3^ In this limit, IVIM is described by a bi‐exponential signal decay, with a diffusion coefficient (D), pseudo‐diffusion coefficient (D*), and perfusion fraction (*f)*. This bi‐exponential IVIM model was used to characterize lesions and monitor treatment response in several studies.^5^, ^6^, ^7^ However, accurate and precise characterization of IVIM remains challenging, because the estimated values of the IVIM parameters depend on acquisition settings^8^ and show large day‐to‐day variations.^9^, ^10^

In the past,^11^, ^12^, ^13^ it was shown that the assumption of the pseudo‐diffusion limit (T/τ<7) is not necessarily valid in abdominal organs and the bi‐exponential signal description is thus inappropriate. This could partially explain the poor reproducibility of *f* and D*, and *f* 's recently reported dependency on blood flow speed.^14^ To overcome these limitations of conventional IVIM, the flow‐compensated (FC) IVIM model was suggested.^13^ In addition to assessing *D* and *f*, FC‐IVIM also models microvasculature with blood flow velocity (*v*) and vessel length (*l*) which are connected through the characteristic time scale τ=l/v. While in conventional IVIM those are combined into $D^{*}=lv/6$, FC‐IVIM allows assessing these additional perfusion‐related parameters, which might be prognostic and predictive for treatment response, separately.

However, fitting the FC‐IVIM model and assessing these microvascular parameters requires additional DWI measurements with FC gradients and at different *T*. Adding those measurements substantially increases the scan time of the already long IVIM acquisition, rendering clinical application unfeasible. For example, the protocol of Wetscherek et al^13^ consisted of 20 breath‐holds of 37.5 s each. In the past, conventional IVIM has successfully been optimized,^15^, ^16^, ^17^ rendering substantially shorter scan protocols; however, there is no guideline to what acquisitions are most important regarding FC‐IVIM.

Therefore, the aim of this work was to develop and test a clinically feasible acquisition protocol for FC‐IVIM in the abdomen by selecting the most informative combinations of *b*, *T*, and gradient profile to enable accurate and precise FC‐IVIM DWI. We achieved this by a simulation study, followed by in vivo validation in healthy volunteers. The resulting optimized protocol facilitates the study of the added clinical benefit of FC‐IVIM.

## 2 METHODS

All data were analyzed in Matlab 2018a (MathWorks, Natick, MA). Figures were created using Matlab and PRISM 8.0 (GraphPad Software, San Diego, CA). The published FC‐IVIM toolbox^13^ was used (downloaded from https://github.com/awetscherek/ivim_tools) for FC‐IVIM modeling and data fitting. Early results from part of this work were presented at the 2018 Joint Annual ISMRM/ESMRMB Meeting.^18^

### 2.1 FC‐IVIM model fitting

We used the following FC‐IVIM model:

(1) $$S\left(b,\;T,\;\beta \right)=S_{0}\;\cdot \;\left[\left(1-f\right)\;\cdot \;e^{-b\;\cdot \;D}+f\;\cdot \;F\left(b,\;T,\;\beta ,\;\tau ,\;v\right)\;\cdot \;e^{-b\;\cdot \;\left(\beta \;\cdot \;D_{b,\mathrm{FC}}+\left(1-\beta \right)\;\cdot \;D_{b,\mathrm{bipolar}}\right)}\right],$$

with

(2) $$F\left(b,\;T,\;\beta ,\;\tau ,\nu \right)=\left|\int_{-\infty }^{\infty }\rho \left(\phi ,\frac{T}{\tau },\;\beta \right)\cdot e^{i\cdot \nu \cdot \sqrt{b\cdot T}\cdot \phi }d\phi \right|.$$

Here, *S* represents the diffusion‐weighted signals, *S*_0_ the signal without diffusion‐weighting, *D* the diffusion coefficient, *f* the perfusion fraction, *v* the blood flow velocity, *τ* the characteristic timescale, *β* the encoding gradient shape (*β* = 0 for bipolar, *β* = 1 for FC), *T* the diffusion time, *ρ* the normalized phase distribution for *β* and the average number of traversed vessel segments =T/τ, and *φ* the normalized phase. *D_b,bipolar_* and *D_b,FC_* are the apparent diffusion coefficients of blood for bipolar and FC gradients, respectively, which we set to values of *D_b,bipolar_* = 1.30 mm^2^/s and *D_b,FC_* = 1.54 mm^2^/s as suggested in literature.^19^ The signal attenuation *F(b*, *T*, *β*, *τ*, *v)* was calculated numerically using the normalized phase distributions included in the FC‐IVIM toolbox.^13^

The model was fitted simultaneously to signal from both FC and non‐FC bipolar measurements with *T* and b‐values using a least squares fit (Matlab's *lsqnonlin* function). The model's fit parameters were *D*, *f*, *v*, *τ*, and the nuisance parameter *S*_0_. Unless mentioned otherwise, fit constraints were set to 0.5 × 10^−3^ < *D* < 3.0 × 10^−3^ mm^2^/s, 0 < *f* < 60%, 20 < τ < 500 ms, 0.2 < v < 15 mm/s.

Per voxel, the signal was normalized to the mean *S*(*b* = 0 mm^2^/s) signal. Initially, a mono‐exponential fit was performed to the high b‐values (*b* ≥ 150 mm^2^/s), of the bipolar gradient data. *D* and *f* from these fits were used to initialize *D* and *f* from the full FC‐IVIM fit. Further fit parameters were initialized with *S*_0_ = 1, τ = 200 ms, and *v* = 4 mm/s.

### 2.2 Simulations

Two sets of simulations were performed. The first determined the best acquisition strategy. The second investigated the influence of signal‐to‐noise ratio (SNR) and perfusion fraction on the accuracy and precision of the estimated FC‐IVIM parameters. In both cases, data were simulated using the FC‐IVIM model equation (Equation 1). Rician noise was added according to the desired SNR and the FC‐IVIM model was fit to the noisy data. We simulated six repeated acquisitions to represent acquisitions from different diffusion directions. These acquisitions were averaged before fitting.

### 2.3 Optimal acquisition strategy

**Figure 1:**
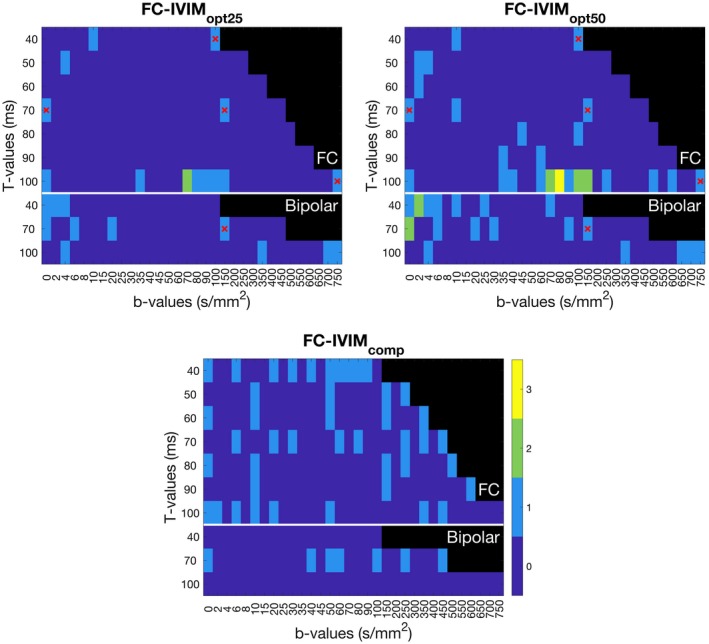
Color coded (dark blue‐yellow, 0‐3 repeated acquisitions) acquisition settings of T‐value (vertical axis), b‐value (horizontal axis), and flow compensation (vertical axis) for four acquisition schemes. Black regions were not accessible on our MR scanner. Images depict the optimal acquisitions when adding 20 (top left) or 45 measurements (top right) to the preselected 5 (red crosses) and the 50 acquisitions of the complementary set (bottom). The order in which measurements were added is provided in Supporting Information Table S1

The optimal acquisition strategy was determined iteratively using an approach similar to Lemke et al,^15^ by repetitively adding the most informative acquisition to the set. A minimum of five acquisitions is required to fit FC‐IVIM, which has five independent variables (*S*_0_, *D*, *f*, *τ*, and *v*); hence, we manually selected the first five acquisition points (red ×'s in Figure 1). Then, we iteratively determined the next most informative (*b*, *T*, *β)* data point to add to the acquisition: given an optimal set of N‐1 acquisitions, the optimal N^th^ acquisition was determined by simulating every possible acquisition 5000 times, and selecting the acquisition that minimized the normalized errors, as detailed below. For these simulations, tissue properties were randomly selected from a predefined range comprising typical abdominal values: *D* = 1‐2 × 10^−3^ mm^2^/s, *f* = 10‐40%, *τ* = 20‐500 ms and *v* = 1‐10 mm/s. Furthermore, Rician noise was added at an effective SNR of 20 at b = 0 s/mm^2^. Considering a range of values for the ground truth parameters prevented the optimal set to be specific to a single parameter combination. The 5000 randomly selected model parameter sets were kept the same for each tested N^th^ acquisition and drawn again for selecting the N+1^th^ one.

Only *b* and *T* combinations that could be experimentally realized on our MRI scanner were tested (nonblack regions in Figure 1). To avoid confounding effects of *T_2_* relaxation,^20^ we chose to keep the echo‐time constant (determined by maximum T). While increasing the maximum T is desirable from a modeling perspective, the consequential increase in TE will reduce the SNR of every measurement due to *T_2_*‐relaxation. Therefore, we limited the range of diffusion times to T_max_ = 100 ms. Values of T lower than T_min_ = 40 ms were not feasible with our hardware. Based on previously published results,^13^ we assumed that the effect of *T* was small for bipolar gradients and we increased the step size for *T* to reduce simulation time. The b‐values ranged from 0 to what was achievable with our gradient set for that given *T* (Figure 1). As most of the flow‐related changes occur at lower b‐values, we used smaller step‐sizes for lower b‐values.

The FC‐IVIM model was fitted to all simulated datasets with constraints: 0 < *D* < 4.0 × 10^−3^ mm^2^/s, 0 < *f* < 100%, 1 < τ < 1000 ms, 0 < v < 20 mm/s). The cost function was determined per added combination of (*b*, *T*, *β)*:

(3) $$cost=\frac{1}{5000}\sum_{n=1}^{5000}\sqrt{\frac{1}{4}\left(D_{error,n}^{2}+f_{error,n}^{2}+\tau_{error,n}^{2}+v_{error,n}^{2}\right)},$$

with

(4) $$\theta_{error,n}=100\%\;\cdot \;\frac{\theta_{true,n}-\theta_{fit,n}}{\theta_{\mathrm{norm}}}.$$

Here, *n* represents the *n* ^th^ experiment and is summed over the 5000 repeats, *θ* can be *D*, *f*, *τ*, and *v*; *θ_error,n_* represents the estimated error in the parameter for the *n* ^th^ experiment; and *θ_true,n_* and *θ_fit,n_* represent the true and estimated values for the *n* ^th^ experiment (for *D*, *f*, *τ*, and *v)*. The errors were normalized by dividing them by *θ_norm_*, which represents the mean parameter value of the interval that the parameter was drawn from (*D*_norm_ = 1.5 × 10^−3^ mm^2^/s, *f*_norm_ = 25%, *τ*_norm_ = 260 ms, and *v*_norm_ = 5.5 mm/s). The acquisition with the smallest mean overall loss was deemed most informative and added as the next measurement. This procedure was repeated 45 times, resulting in selecting the optimal 45 (+5 initial) acquisition points.

The performance of FC‐IVIM at each added acquisition was simulated a second time using a new random selection of simulation parameters to prevent performance bias by selection of acquisition with minimal error. These simulations used the new, earlier mentioned fit constraints. The error per parameter, *θ_error,n_*, was calculated using Equation 4. The 5, 25, 50, 75, and 95 percentiles of *θ_error,n_* were plotted to study the relation between acquisition time and expected error.

### 2.4 Minimum SNR and f

To accurately estimate the FC‐IVIM model parameters describing the vasculature (*f*, *τ*, and *v*), sufficient SNR and blood signal (f) needs to be present. Hence, simulations were done to investigate the relation between SNR and *f*, and the accuracy and precision of the estimated FC‐IVIM parameters. The optimal 50 acquisitions from the previous simulations (Figure 1, top right) were used as simulated acquisition protocol. For both simulation series, 1 parameter was varied (SNR: 10‐60; *f*: 3‐50%) while having the rest set to literature values^13^: *D* = 1.88 × 10^−3^ mm^2^/s, *f* = 28.7%, *τ* = 224 ms, and *v* = 3.91 mm/s for pancreas and *D* = 1.12 × 10^−3^ mm^2^/s, *f* = 34.7%, *τ* = 144 ms, and *v* = 4.60 mm/s for liver. SNR was set to 20 for the simulations varying *f*. Simulations of fits were repeated 5000 times while adding new random noise each time. The fit error was determined as defined in Equation 4, with $\theta_{\mathrm{norm}}=\theta_{true,n}=\theta_{\mathrm{true}}$. These simulations were repeated with a manually selected set of 50 complementary (*b*, *T*, *β)* values (Figure, 1 bottom), to highlight the gain from selecting an optimized acquisition scheme over a naive one. The 5, 25, 50, 75, and 95 percentiles of $\theta_{error,n}$ were plotted.

### 2.5 In vivo measurements

Abdominal DWI images of seven healthy volunteers (three female, four male; aged 21‐28 years old) were obtained between August and November 2018 using a 1.5T scanner (Magnetom Aera, Siemens Healthineers) at the University Hospital Erlangen. The local ethics committee of the University Hospital Erlangen approved volunteer scanning and all volunteers gave written informed consent. Scans were obtained with a 30‐channel anterior body coil and 32‐channel posterior coil in the table. The scanner was equipped with XQ gradients (G_max_ = 45 mT/m, S_max_ = 200 mT/m/ms). The DWI acquisitions were repeated during the first session to enable assessment of intrasession repeatability, and a second session was held 7 days (median, 7 days; range, 6‐14 days) later to enable the assessment of intersession repeatability. While the intrasession repeatability characterizes short‐term accuracy, the intersession repeatability additionally accounts for day‐to‐day and setup‐related variation.

DWI data were acquired during free breathing, without motion management, using a diffusion‐weighted single‐shot echo planar imaging read‐out. The acquisition consisted of six 8‐mm‐thick slices, acquired in descending order, with 8‐mm slice gap to prevent inflow of recently exited blood from neighboring slices. Field of view was 284 × 350 mm^2^, with 2.7 × 2.7 mm^2^ voxel size. Other settings were: repetition time = 3000 ms; echo time = 122 ms; partial Fourier factor = 6/8; and acceleration factor = 2 (GRAPPA, 24 reference lines); SPAIR fat suppression; bandwidth 1775 Hz/pixel; diffusion gradients were applied simultaneously on two gradient axes along six directions (multi‐directional diffusion weighting: [1, 1, 0], [1, −1, 0], [1, 0, 1], [1, 0, − 1], [0, 1, 1], and [0, 1, − 1]). The acquisition consisted of the five base (*b*, *T*, *β)* values (red crosses in Figure 1), the 45 most informative (*b*, *T*, *β)* values according to the simulations (Figure 1, top right), and an acquisition of 50 manually selected complementary (*b*, *T*, *β)* values (Figure 1, bottom). To make implementation easier, the optimal acquisition set was limited to T = 40, 70, and 100 ms and the seven measurements of other T were moved to the closest acquired T (Supporting Information Table S1, which is available online, gives the implemented acquisition). Acquisition of the sets took 32 min each (optimized and complementary), resulting in a scan time of 64 min for the first session, which included one repetition and 32 min for the second session.

### 2.6 Signal preprocessing

Per DWI dataset, for each slice, a 2D+temporal principal component analysis (PCA) based groupwise image registration^21^ was performed in Elastix^22^ to register the images from the different sets and (*b*, *T*, *β*)‐values to each other. For contouring one purposes, registered images were denoised using PCA^23^ followed by maximum intensity projection over the six acquisition directions^24^ (using the PCA denoising method from Gurney‐Champion et al^23^). A researcher with six years of experience in abdominal imaging (O.J.G.) then contoured the liver, spleen, and both kidneys on all slices that were within the acquired field of view for both imaging sessions. The regions of interest (ROIs) were propagated to the repeated intersession and intrasession images using rigid image registration and updated manually where required. ROIs had a median size (range) of 4592 (2590‐6032) voxels, 945 (344‐1360), 733 (488‐987), and 437 (301‐718) voxels for the liver, spleen, left kidney, and right kidney, respectively.

Due to the long acquisitions, potentially signal drifts occurred as a result of, e.g., gradient heating.^25^ Hence, per contour, all acquisitions with *b* = 0 s/mm^2^ and their corresponding acquisition times were selected and Matlab's *fitlm* was used to detect potential drifts in the mean signal intensity from that ROI. The function returned an offset, *a*, and slope, *b*. If the slope was significantly nonzero (*P* < 0.05), the data from that ROI was corrected accordingly, (assuming linear scaling) using

(5) $$S_{\mathrm{corrected}}\left(t\right)=\frac{S_{\mathrm{old}}\left(t\right)}{a+b\;\cdot \;t}$$

Here, *S_corrected_* is the corrected signal and *S_old_* the original signal.

### 2.7 Analysis

Four FC‐IVIM fits were performed to the median signal decay in each ROI using the measurements at (I) the 5 base + 20 optimized measurements, (II) the 5 base + 45 optimized measurements, (III) the 50 complementary measurements and (IV) all 100 measurements combined, which we will call FC‐IVIM_opt25_, FC‐IVIM_opt50_, FC‐IVIM_comp_, and FC‐IVIM_100_, respectively. Organ‐specific fit constraints, as described in Table 1, were used. Fit quality was assessed by the within‐subject coefficient of variation (*wCV*) for the repeated measures. More stable measurements, at informative (*b*, *T*, *β*) are expected to be more precise and, hence, have a lower *wCV*.

**Table 1:** Organ‐specific fit constraints

	Liver		Spleen		Kidneys	
	Min	Max	Min	Max	Min	Max
*D* (10^−3^ mm^2^/s)	0.6	2.5	0.5	1.5	1.0	3.0
*f* (%)	20	80	5	25	10	35
*τ* (ms)	20	500	50	300	20	500
*v* (mm/s)	0.2	12	0.2	12	3	15

Another quantity assessing data reliability from repeated measurements is the two‐way mixed effects, absolute agreement, multiple measurements intraclass correlation coefficient (*ICC*).^26^, ^27^ We calculated the *wCV* and *ICC* for the optimal acquisition scheme (FC‐IVIM_opt50_). *ICC* values of above 0.75 were considered to have good reliability.

In vivo, the ground truth is unknown. By assuming that the FC‐IVIM_100_ data, which contains all measured (*b*, *T*, *β*) combinations, allows for the best estimate of the true parameters, the bias of the other acquisition schemes can be estimated by taking the mean of the normalized difference:

(6) $$bias_{\theta }=100\%\;\cdot \;\frac{1}{M}\sum_{m=1}^{M}\frac{\theta_{\mathrm{FC}-{\text{IVIM}}_{100},m}-\theta_{\mathrm{FC}-{\text{IVIM}}_{opt50},m}}{\theta_{\mathrm{FC}-{\text{IVIM}}_{100},m}}.$$

Here, $\theta_{IVIM_{100},m}$ and $\theta_{IVIM_{opt50},m}$ are the parameters (*D*, *f*, *τ*, and *v*) of FC‐IVIM_100_ and FC‐IVIM_opt50_ from the m^th^ volunteer and *M* the total number of volunteer measurements. This was repeated for IVIM_comp_, where $\theta_{\mathrm{FC}-{\text{IVIM}}_{opt50},m}$ is replaced by $\theta_{\mathrm{FC}-{\text{IVIM}}_{\mathrm{comp}},m}$ in Equation 6.

## 3 RESULTS

### 3.1 Simulations

Figure 1 illustrates the initial 20 and 45 selected measurements, whereas Supporting Information Table S1 shows the order in which the measurements were added. In general, several measurements were added at high T (100 ms) with midrange b‐values (60‐150 s/mm^2^) for the FC gradients, whereas for bipolar gradients low b‐values were preferred (0‐30 s/mm^2^). These simulations took two months running on 5 cores of a 3.5 GHz Intel Core i7‐4771.

**Figure 2:**
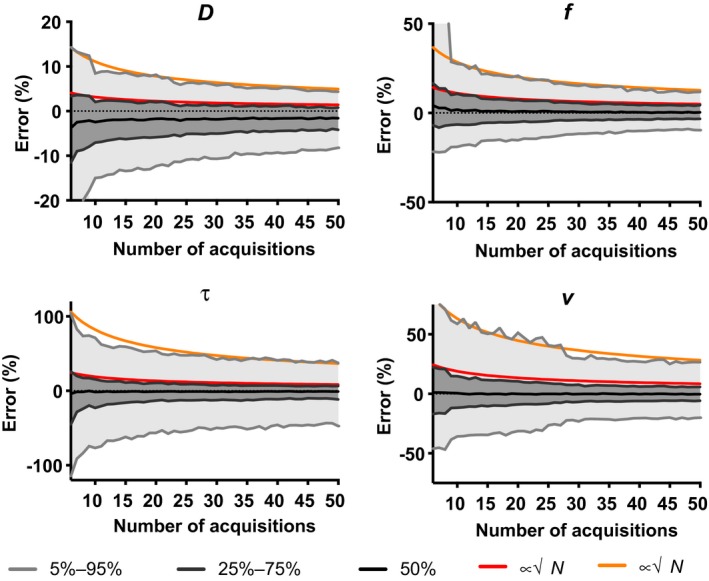
The error of *D*, *f*, *τ*, and *v* as a function of the number of optimized acquisitions. The 5%‐95% interval (light gray), 25%‐75% (dark gray) interval, and median value of the 5000 repeated simulations are plotted. The corresponding asymptotic $\sqrt{N}$ limit is displayed in orange and red, respectively, where *N* denotes the number of acquisitions

Figure 2 illustrates how the errors decreased as a function of the number of selected optimal acquisitions. The plotted red and orange lines show that the reduction was roughly proportional to √*N*. We found that the largest error reduction occurred within the first 25 measurements. However, increasing the number of acquisitions from 25 to 50 did still substantially decrease the error and would be desired if measurement time was available.

**Figure 3:**
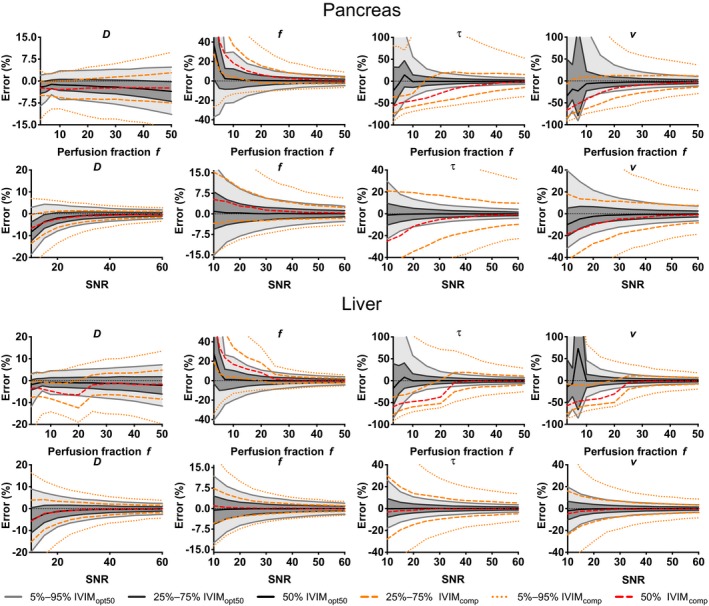
Plots of the error as a function of SNR and *f* for the liver (bottom) and pancreas (top) parameters. The 5%‐95% interval (light gray), 25%‐75% (dark gray), interval and median (black) value were plotted for the 5000 repeated simulations of the IVIM_opt50_ acquisition. The 5%‐95% and 25‐75% intervals (orange dashed) and median (red dashed) values of the 5000 repeated simulations of the IVIM_comp_ were plotted for comparison

At optimal settings, simulated tissue with larger *f* gave substantial smaller errors in *f*, *v* and *τ*, whereas the error in *D* increased slightly. All errors decreased as a function of SNR (Figure 3). The behaviors above were similar for both simulated datasets (pancreas and liver), which suggests this is a general trend. IVIM_opt50_ (Figure 3, gray shades) outperformed IVIM_comp_ (Figure 3, orange and red dashed lines) consistently. The graph demonstrated, for example, that for the liver, at an SNR of 20, we expect to estimate D, *f*, *v*, and *τ* with the 5%‐95% percentiles at boundaries −8.4%‐5.9%, −6.1%‐6.2%, −10.5%‐10.2%, and −9.4%‐10.6% for IVIM_opt50_. For IVIM_comp_, these boundaries are considerably broader, at −15.8%‐10.2%, −7.4%‐10.0%, −29.9%‐26.2%, and −35.9%‐44.8%.

### 3.2 In vivo

**Figure 4:**
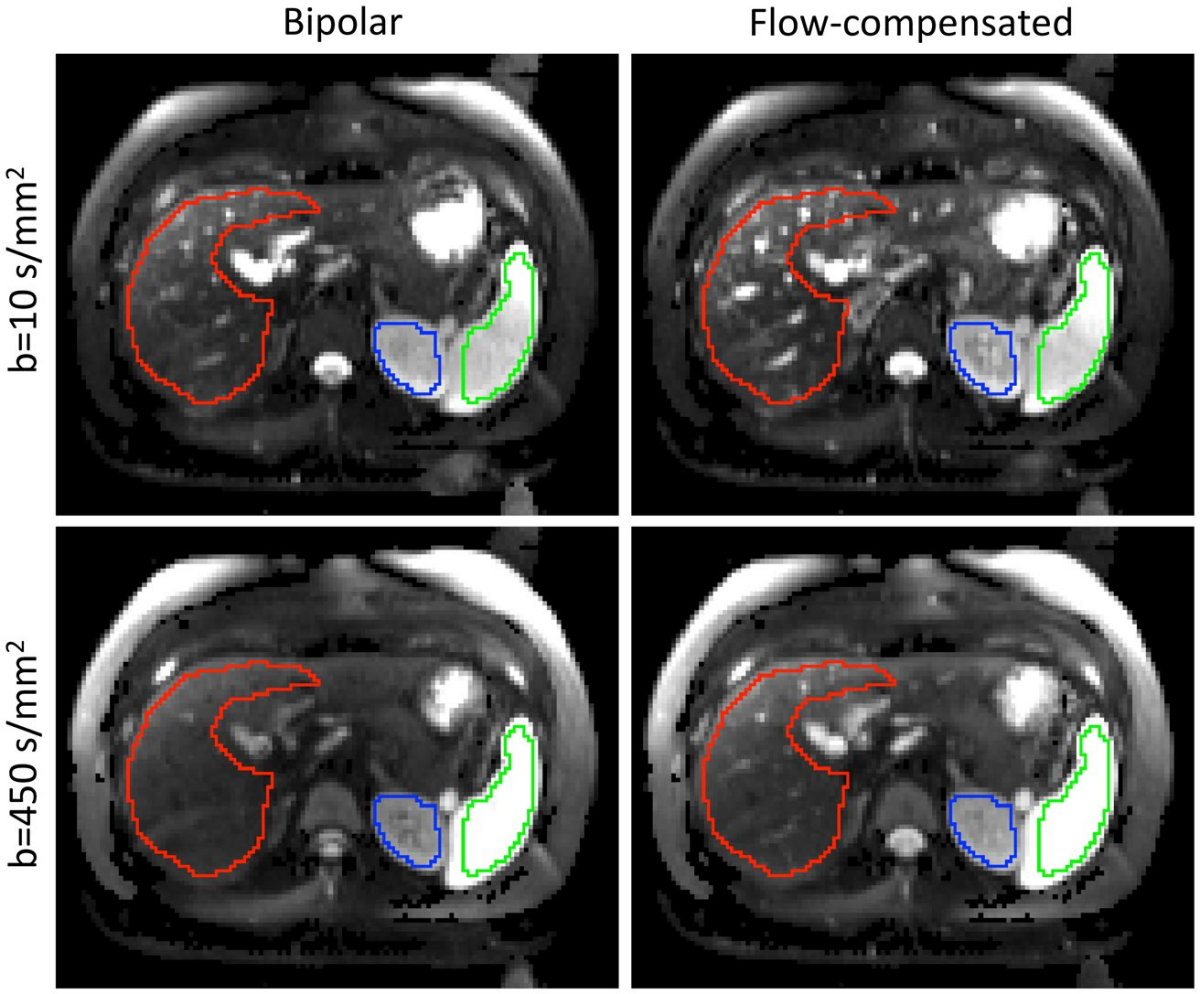
Example images (after registration, PCA denoising, and maximum intensity projection) of b = 10 s/mm^2^ and b = 450 s/mm^2^ acquired with bipolar (left) and FC (right) gradients. Note that signal decay in vessels is less apparent in the FC scheme. ROIs: red, liver; green, spleen; blue, kidney

**Figure 5:**
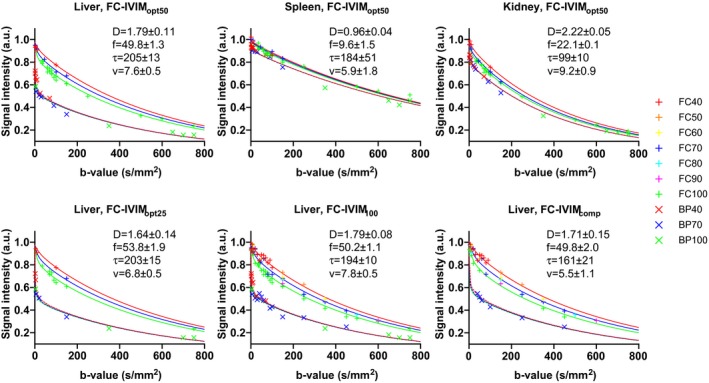
Representative fits to data from all investigated acquisition approaches (FC‐IVIM_opt25_, FC‐IVIM_opt50_, FC‐IVIM_100_, and FC‐IVIM_comp_) for the liver, as well as plots of data from all evaluated organs at the optimal approach FC‐IVIM_opt50_. The +'s indicate FC data at different *T*, for which the fit result is described by solid lines. The ×’s indicate the data from bipolar gradients at different *T*, for which the fit result is described by the overlapping dashed lines. The units of the fitted values are: *D* [10^−3^ mm^2^/s], *f* [%], *τ* [ms], and *v* [mm/s]

Figure 4 illustrates the image quality of the processed data and delineated ROIs from a representative volunteer. Figure 5 shows typical fits of the FC‐IVIM model to ROI‐averaged data. Exemplary fits for each organ at optimal settings (liver, spleen, and kidneys using FC‐IVIM_opt50_) are displayed to highlight the variety in signal decays. Furthermore, the data from different acquisition approaches (FC‐IVIM_opt25_, FC‐IVIM_opt50_, FC‐IVIM_100_, and FC‐IVIM_comp_ for the liver) are shown for the liver. Table 2 reports the median (over different organs) intersession and intrasession *wCV*s of the FC‐IVIM parameters for the different imaging approaches and illustrates that FC‐IVIM_opt50_ performs best compared with the similarly long FC‐IVIM_comp_ acquisition.

**Table 2:** Median (over all organs) intersession and intrasession *wCV*s (%) of the different fit parameters for the different datasets

Intersession	FC‐IVIM_opt25_	FC‐IVIM_opt50_	FC‐IVIM_100_	FC‐IVIM_comp_
*D*	12.7	11.1	11.8	15.9
*f*	29.2	16.3	16.0	25.5
*τ*	62.1	65.5	39.2	57.8
*v*	64.0	36.0	26.5	64.5
**Intrasession**				
*D*	16.4	9.4	5.9	16.2
*f*	36.5	14.1	12.9	16.7
*τ*	83.7	71.2	30.3	62.1
*v*	89.0	56.2	42.4	123.9

Supporting Information Table S2 reports the intersession and intrasession *wCV*s for all ROIs. FC‐IVIM_100_ (32‐min acquisition) had the highest precision, with intersession *wCV* lower than *wCV* of FC‐IVIM_opt50_ in 11 of 16 comparisons (4 organs × 4 parameters). FC‐IVIM_opt50_ showed the best results of all protocols obtainable within 16 min. FC‐IVIM_opt50_ had a lower intersession *wCV* than FC‐IVIM_comp_ for 14 of 16 comparisons and had lower intersession *wCV* than FC‐IVIM_opt25_ for 12 of 16 comparisons. The intersession *wCV*s of FC‐IVIM_opt50_ were on average 29% lower than the intersession *wCV* of FC‐IVIM_comp_ [100% × 2 × (*wCV*_comp_−*wCV*_opt50_)/ (*wCV*_comp_+*wCV*_opt50_)] and 23% lower than IVIM_opt,25_. The intersession *wCV*s of FC‐IVIM_100_ (32‐min acquisition) were on average 19% lower than the *wCV* of FC‐IVIM_opt50_ (16‐min acquisition). Compared with FC‐IVIM_comp_, FC‐IVIM_opt25_ (8‐min acquisition) had a lower intersession *wCV* in 9 of 16 comparisons, with intersession *wCV* on average 6% higher. The intrasession *wCV* was smaller than the intersession *wCV* in only 26 of 64 comparisons (four parameters, four organs, four acquisition schemes).

Table 3 shows that the estimated bias in the parameter estimates from FC‐IVIM_opt50_ are smaller than the estimated bias in the parameter estimates from FC‐IVIM_comp_.

**Table 3:** Estimated bias expressed as mean of the % difference from FC‐IVIM_opt50_ and FC‐IVIM_comp_ compared to FC‐IVIM_100_ (Equation 6)

FC‐IVIM_opt50_	Liver	Spleen	Left kidney	Right kidney
*D* (%)	−4.6	−0.4	−1.0	0.0
*f* (%)	−1.7	0.2	−2.8	−1.9
*τ* (%)	−1.4	16.5	−1.3	8.5
*v* (%)	5.0	3.0	11.6	6.0
**FC‐IVIM_comp_**				
*D* (%)	−6.9	0.7	−1.0	−0.9
*f* (%)	−7.8	2.1	6.3	5.0
*τ* (%)	−15.1	9.0	−13.7	8.7
*v* (%)	−27.1	4.2	−21.8	−11.4

The *wCV*s and *ICC*s at optimal setting (FC‐IVIM_opt50_) are reported in Table 4. *D* and *f* had good reliability (*ICC* ≥ 0.75) in seven of eight measures and *v* and *τ* had worse reliability (*ICC* < 0.75) in all measures. This is in agreement with the *wCV*s being generally higher for *f* and *D*. Table 5 shows the mean fitted parameter values and their standard deviation over the different volunteers (including the three repeated measurements) for these optimal settings (FC‐IVIM_opt50_). The spleen had lowest perfusion signal fraction (Table 5: 9.3 ± 2.7 %) and poorest *wCV* for the perfusion‐related parameters (Table 4: *wCV* of *f* is 56.7%; *wCV* of *τ* is 124.7%; *wCV* of *v* is 143.2%), whereas the liver had the highest perfusion signal fraction (45.0 ± 4.7 %) and best *wCV* for the perfusion‐related parameters (e.g., *wCV* of *f* is 14.7%; *wCV* of *τ* is 25.2%; *wCV* of *v* is 12.5%).

**Table 4:** FC‐IVIM_opt50_ intersession *wCV*s (%; top half) and ICCs (bottom half) of the different organs

*wCV*	Liver	Spleen	Left kidney	Right kidney
*D*	17.2	10.1	12.2	9.0
*f*	14.7	56.7	14.8	17.8
*τ*	25.2	124.7	71.3	59.7
*v*	12.5	143.2	25.8	46.1
**ICC**				
*D*	0.75	0.92	0.79	0.81
*f*	0.90	0.46	0.88	0.77
*τ*	0.50	n.a.	0.57	0.40
*v*	0.74	n.a.	0.70	0.63

Note

that the ICC of *τ* and *v* were negative for the spleen due to a large within‐subject variability and hence are unreliable (n.a.).

**Table 5:** Mean parameter values ± standard deviation (SD) over the volunteers using our optimized FC‐IVIM_opt50_ protocol

FC‐IVIM_opt50_	Liver		Spleen		Left kidney		Right kidney	
	Mean	SD	Mean	SD	Mean	SD	Mean	SD
*D* (10^−3^ mm^2^/s)	1.61	±0.16	0.84	±0.07	2.30	±0.16	2.36	±0.14
*f* (%)	45.0	±4.7	9.3	±2.7	19.4	±1.9	19.6	±2.3
*τ* (ms)	171	±19	147	±54	71	±22	71	±19
*v* (mm/s)	8.4	±0.6	5.9	±2.8	10.8	±1.5	10.8	±2.1

## 4 DISCUSSION

We developed an acquisition protocol for FC‐IVIM in the abdomen with a clinically feasible scan time (8 and 16 min for our two suggested protocols). This was achieved by making use of simulations to select the most informative combinations of b, T, and gradient shape to obtain accurate and precise FC‐IVIM DWI. We then showed that our protocol outperforms naive protocols of similar acquisition times in simulations and in abdominal scans of healthy volunteers. The use of our suggested protocol results in accurate and precise estimations of *D* and *f*, whereas the accuracy of the microscopic FC‐IVIM parameters *v* and *τ* was similar to the accuracy of D* from conventional non‐FC IVIM.^16^ This is the first study to determine the optimal scanning protocol for FC‐IVIM and the first which examines the test‐retest precision of FC‐IVIM parameters. Our results demonstrate the feasibility of implementing FC‐IVIM in clinical studies and provide data on precision to inform power calculations.

We find that *f* is estimated at high precision, particularly for the liver (intersession *wCV* = 14.7%) and kidneys (intersession *wCV* = 14.8‐17.8%) when compared with literature (liver: 11.4‐34%^16^, ^28^, ^29^, ^30^, ^31^ median 25.3; kidney: 25‐36%^32^). The *wCV* of *D* for FC‐IVIM was similar to literature values of conventional IVIM (*wCV* D = 5‐17%^16^, ^29^, ^30^, ^31^, ^32^, ^33^). The *wCV* of v and *τ* from FC‐IVIM are in a similar range as D* from conventional IVIM model (e.g., 25‐194 for various abdominal organs^16^, ^29^, ^30^, ^31^, ^32^, ^33^).

Recently, it was also shown in phantom measurements that FC‐IVIM better reflects the actual microstructure than conventional IVIM: only FC‐IVIM was able to estimate f accurately and precisely, independent of the applied blood flow.^14^ The combination of the improved description of physiology, together with the improved accuracy and precision, clearly highlights the potential advantages of FC‐IVIM over conventional IVIM.

To find the optimal acquisition in the simulations, all errors were deemed equally important. Therefore, the added acquisitions mainly focused on reducing the error in the parameters with the largest errors, i.e., *τ* and *v*, whereas the errors in *D* and *f* decreased slower (Figure 2). One could add weights to the different parameters when determining the overall error in the simulation to focus on optimizing the sequence for certain parameters. As FC‐IVIM has not been investigated in patients and hence clinically relevant effect‐size is unknown, it is unclear whether these values are clinically sufficient or whether certain parameters should receive higher weights during optimization.

We focused on abdominal imaging; however, we believe our methods and results can be useful for other tissue too. Our simulations were performed for a broad range of tissue parameters to ensure our protocol was not optimized to a very specific parameter value. Organs with tissue properties that fall within this range should also benefit from the optimized protocol too. The brain generally has a lower perfusion fraction (f~5%) than our simulation range,^34^ as well as typically higher SNR. To find optimal settings in brain MRI, simulations could be repeated with different settings.

FC‐IVIM performs better at higher perfusion fractions. This is initially shown in our simulations (Figure 3) and confirmed in our in vivo data. In vivo, the ROI with highest *f*, the liver (*f* = 45.0 ± 4.7%), had overall best precision for the perfusion‐related parameters and the ROI with lowest *f*, the spleen (*f* = 9.3 ± 2.7%), had overall worst precision for the perfusion‐related parameters. Furthermore, our simulations suggest that higher SNR increases the performance. We would, therefore, suggest initially implementing FC‐IVIM clinically in well‐perfused organs with high SNR.

As the intrasession *wCV* is only affected by short‐term (< 60 min) variations in the parameters, whereas the intersession *wCV* is additionally affected by day‐to‐day variations, one would expect intrasession *wCV* to be smaller than or equal to the intersession *wCV*. There are several explanations why, for our dataset, the intrasession *wCV* was similar to the intersession *wCV*. Long‐term biological variations could be smaller than short‐term variations for the diffusion and perfusion processes measured, resulting in similar *wCV*s, both dominated by the short‐term contribution. Furthermore, the general measurement accuracy could be of the order of the long‐term and short‐term biological variations, and hence dominate the uncertainty in both cases. Finally, the sample size of volunteers could be too small to detect differences between intersession *wCV* and intrasession *wCV*.

There is still debate on the use of motion management in IVIM, with conflicting advice.^31^, ^35^ This is reflected in the ISMRM consensus on DWI outside the brain, where for IVIM “a free‐breathing or respiratory‐triggered” protocol is advised.^36^ As our purpose was to compare the optimized sequence to a nonoptimized complementary sequence, many diffusion‐weightings (100 for intersession, 200 for intrasession) had to be obtained per session. To achieve this within a single scan session, no respiratory motion management was performed. We have taken multiple steps in minimizing the effect of motion and believe it did not influence our results. We performed image registration to ensure images are aligned. Furthermore, most of our gradients were FC gradients that are typically used in cardiac imaging for their motion robustness.^37^ Moreover, we had slice gaps to prevent inflow of excited blood and cross‐talk between slices. Finally, we used PCA and maximum intensity projection^24^ to correct for any remaining motion‐related signal dropout.

In this study, FC‐IVIM fits were performed ROI‐wise, whereas voxel‐wise fits might be preferred for clinical applications. With the current implementation, voxel‐wise fits take long (seconds per voxel) and are susceptible to noise and partial voluming effects. For conventional IVIM, several attempts have been made on optimizing voxel‐wise fits using Bayesian approaches,^38^, ^39^ segmented fits,^40^ non‐negative least squares,^41^ and, recently, neural‐network‐based fit approaches.^42^, ^43^ To render future FC‐IVIM applications with voxel‐wise parameter estimation possible, fitting approaches with improved robustness should be investigated.

Only one study in literature has investigated FC‐IVIM parameters in the abdomen,^13^ where *D* = 1.12 × 10^−3^ mm^2^/s, *f* = 34.7%, *τ* = 144 ms, and *v* = 4.6 mm/s were observed in the liver. In contrast, we observed *D* = 1.61 ± 0.16 × 10^−3^ mm^2^/s, *f* = 20 ± 4.7%, *τ* = 171 ± 19 ms, and *v* = 8.4 ± 0.6 mm/s. We believe the difference in parameter values might be caused by different strategies for delineating the liver: Wetscherek et al^13^ avoided larger vessels, as done in conventional IVIM. In conventional IVIM, *D* ranges from 0.66‐1.50 × 10^−3^ mm^2^/s and *f* from 5.5‐47.1% in literature.^44^ The relatively large *f* that we report is a result of the long echo time, which decreases the signal contribution of liver tissue, with short T_2_ of typically 46 ± 6 ms,^45^ compared with venous blood, with T_2_ in the order of 181 ± 23 ms.^46^ Assuming these T_2_‐values, our *f* would have been 20.3%^20^, ^47^ if we had a typical TE of 50 ms. This value agrees well with the literature range.

For the spleen, we found a mean *D* of 0.84 ± 0.07 × 10^−3^ mm^2^/s, which is in the same range as reported by Jerome et al, who found *D* = 0.91 ± 0.16 × 10^−3^ mm^2^/s in the spleens of 10 healthy volunteers.^35^ We found an *f* = 9.3 ± 2.7%, which is between the literature values of *f* = 7.6%^35^ and *f* = 12%.^48^ We found *D* = 2.30 ± 0.16 × 10^−3^ mm^2^/s and 2.36 ± 0.14 × 10^−3^ mm^2^/s, and *f* = 19.4 ± 1.9% and 29.6 ± 2.3% for the left and right kidneys, respectively. These values are within the range of reported literature values in healthy volunteers of *D* = 1.4‐2.1 × 10^−3^ mm^2^/s and *f* = 9‐33%.^32^, ^49^, ^50^, ^51^ Recently, a T_2_‐dependence of *D* was reported in prostate cancer patients,^52^ which could point to our long echo times as a possible explanation why our D‐values lay at the edges of the interval of reported literature values. Another factor influencing the values could be magnetic field strength.^53^

A limitation of this study was the broader fit boundaries used to determine the optimal parameters. Later on in the research project, we found that tighter boundaries resulted in a more stable fit. However, volunteer data were already acquired with the optimal set determined with broader fit boundaries. By repeating the simulations (results not shown), we found tighter fit boundaries resulted in similar optimal acquisition distribution. Another limitation is the manually chosen initial five acquisition points. The results in figure 1 would indicate that these acquisition points are not necessarily representative of the combinations selected by the algorithm. Another limitation was the large slice gap (8 mm) we used to prevent the inflow of excited blood and crosstalk between slices. Potentially, interleaved scanning with longer TRs could overcome this limitation, but this would add substantially to the scanning time. Cross‐talk and, in particular, inflow of blood that was exited in previous slice acquisitions is a challenge in general IVIM measurements which deserves further investigation. Although DWI was obtained in six different diffusion directions, the effect of diffusion direction on the signal was not investigated in this study, as it is not considered in the FC‐IVIM model.

The FC‐IVIM protocol was only studied in young and healthy volunteers. To date, no FC‐IVIM protocol, including our 1‐h sessions, was compact enough for patient scanning. Our optimized protocol is now compact enough to scan in patients. As our protocol does not rely on requirements that would be different in a patient (such as the ability to follow breathing instructions), we believe its performance will be similar to the results from this work. If 8 min is still considered too long an acquisition, the optimal b‐values for shorter acquisitions can be deduced from Supporting Information Table S1 and the performance is shown in Figure 2.

## 5 CONCLUSIONS

In this study, we demonstrated the feasibility of including FC‐IVIM in a clinical setting to investigate changes in vasculature in strongly perfused organs. We have optimized the FC‐IVIM acquisition characterizing the accuracy (simulations) and precision (simulations and in vivo experiments) of FC‐IVIM parameter estimates. The resulting optimized acquisition strategies (FC‐IVIM_opt25_ and FC‐IVIM_opt50_) facilitate clinically acceptable acquisition times (8‐16 min). At the optimal settings, FC‐IVIM shows better test‐retest precision than conventional IVIM, particularly for estimating the perfusion fraction. FC‐IVIM is now ready to be used in clinical trials, to assess its value.

## Supporting information


**TABLE S1** The order of the selected ideal b, T, and β values
**TABLE S2** Intersession (inter) and intrasession (intra) *wCV*s (%) of the different organs
