# Supplementary material for: Optimal acquisition scheme for flow‐compensated intravoxel incoherent motion diffusion‐weighted imaging in the abdomen: An accurate and precise clinically feasible protocol
Source: Magn Reson Med. 2019 Sep 30;83(3):1003–15. doi: 10.1002/mrm.27990 (PMC6899942; doi:10.1002/mrm.27990)
Supplement: Supplementary file 1 — TABLE S1 The order of the selected ideal b, T, and β values TABLE S2 Intersession (inter) and intrasession (intra) wCVs (%) of the different organs [file MRM-83-1003-s001.pdf]

Supporting Information

Supporting Information Table S1: Selected ideal b, T and β are indicated in the left columns and, where deviated, the actual acquired setting is indicated in the right column.

	Optimal settings			Acquired		
	b (10^{-3} mm^2/s)	T (ms)	β	b (10^{-3} mm^2/s)	T (ms)	β
	0	70	1			
	100	40	1			
	750	100	1			
	150	70	1			
	150	70	0			
1	2	40	0			
2	70	100	1			
3	80	100	1			
4	6	70	0			
5	700	100	0			
6	0	40	0			
7	100	100	1			
8	70	100	1			
9	20	70	0			
10	0	100	1			
11	35	100	1			
12	10	40	1			
13	150	100	1			
14	4	100	0			
15	90	100	1			
16	350	100	0			
17	4	50	1	4	40	1
18	750	100	0			
19	0	70	0			
20	4	40	0			
21	30	70	0			
22	150	100	1			
23	2	40	0			
24	80	100	1			
25	25	40	0			
26	6	40	0			
27*	2	60	1	2	70	1
28	600	100	1			
29*	45	80	1	45	70	1

30*	2	50	1	2	40	1
31*	100	80	1	100	70	1
32	40	100	1			
33	90	70	0			
34	80	100	1			
35	60	100	1			
36	100	100	1			
37*	60	90	1	60	100	1
38	70	40	0			
39	650	100	0			
40	0	70	0			
41	10	40	0			
42*	35	90	1	35	100	1
43	10	70	1			
44	500	100	1			
45	250	100	1			

Supporting Information Table S2: Intersession (inter) and intrasession (intra) wCVs (%) of the different organs.

	**Liver**		**Spleen**		**Left kidney**		**Right kidney**	
**FC-IVIM$_{opt25}$**	Inter	Intra	Inter	Intra	Inter	Intra	Inter	Intra
D	26.8	18.2	12.5	34.4	11.0	10.0	12.9	14.7
f	24.2	14.4	41.6	89.2	20.5	40.2	34.2	32.8
τ	35.2	25.59	129.6	100.1	34.9	75.6	89.0	91.7
v	26.2	32.3	102.9	172.2	60.2	96.2	67.8	81.9
**FC-IVIM$_{opt50}$**								
D	17.2	9.7	10.1	16.4	12.2	3.9	9.0	9.2
f	14.7	13.7	56.7	21.8	14.8	14.5	17.8	13.4
τ	25.2	18.2	124.7	90.9	71.3	86.7	59.7	55.6
v	12.5	13.4	143.2	76.4	25.8	47.3	46.1	65.1
**FC-IVIM$_{100}$**								
D	17.4	9.0	4.7	14.5	13.0	2.7	10.7	2.7
f	14.6	12.4	58.0	54.2	12.8	13.4	17.4	7.3
τ	17.7	14.6	101.4	80.6	38.5	37.3	40.0	23.3
v	20.2	19.3	87.0	131.2	13.2	36.9	32.9	48.0
**FC-IVIM$_{comp}$**								
D	19.7	33.0	16.8	24.6	15.0	7.8	11.5	5.0
f	17.0	19.2	121.4	125.5	23.7	14.0	27.2	14.2
τ	37.6	76.3	163.6	129.5	52.7	35.5	62.9	47.9
v	50.8	220.7	91.6	179.7	28.0	64.4	74.1	68.0

Supporting Information to " Optimal acquisition scheme for flow-compensated intravoxel incoherent motion diffusion-weighted imaging in the abdomen: an accurate and precise clinically feasible protocol" by Gurney-Champion et al., MRM. 2019
